# DOT1-like histone lysine methyltransferase is critical for adult vessel maintenance and functions

**DOI:** 10.5713/ab.23.0402

**Published:** 2024-04-24

**Authors:** HeeJi Lee, Dong Wook Han, Hyeonwoo La, Chanhyeok Park, Kiye Kang, Ohbeom Kwon, Sang Jun Uhm, Hyuk Song, Jeong Tae Do, Youngsok Choi, Kwonho Hong

**Affiliations:** 1 Department of Stem Cell and Regenerative Biotechnology, Institute of Advanced Regenerative Science, Konkuk University, Seoul 05029, Korea; 2 Guangdong Provincial Key Laboratory of Large Animal Models for Biomedicine, Wuyi University, Jiangmen 529020, China; 3 Department of Animal Science, Sangji University, Wonju 26339, Korea

**Keywords:** Blood Endothelial Cell, DOT1L, Hemorrhage, Transcriptional Regulation

## Abstract

**Objective:**

Disruptor of telomeric silencing 1-like (DOT1L) is the only known histone H3K79 methyltransferase essential for the development of the embryonic cardiovascular system, including the heart, blood vessels, and lymphatic vessels, through transcriptional regulation. Our previous study demonstrated that Dot1l deletion results in aberrant lymphatic development and function. However, its precise function in the postnatal cardiovascular system remains unknown.

**Methods:**

Using conditional and inducible Dot1l knockout (KO) mice, along with a reporter strain carrying the Geo gene at the Dot1l locus, DOT1L expression and its function in the vascular system during postnatal life were investigated. To assess vessel morphology and vascular permeability, we administered Latex or Evans blue dye to KO mice. In addition, in vitro tube formation and cell migration assays were performed using DOT1L-depleted human umbilical vein endothelial cells (HUVECs). Changes in the expression of vascular genes in HUVECs were measured by quantitative polymerase chain reaction.

**Results:**

Our findings demonstrate that conditional Dot1l knockout in the Tg (Tie2-cre) strain results in abnormal blood vessel formation and lymphatic anomalies in the intestine. In a mouse model of Rosa26-creER-mediated inducible Dot1l knockout, we observed vascular phenotypes, including increased vascular permeability and brain hemorrhage, when DOT1L was deleted in adulthood. Additionally, DOT1L depletion in cultured HUVECs led to impaired cell migration and tube formation, likely due to altered gene transcription. These findings highlight the essential role of DOT1L in maintaining vascular integrity and function during embryonic development and postnatal life.

**Conclusion:**

Our study revealed that DOT1L is required for the maintenance of adult vascular function through the regulation of gene expression.

## INTRODUCTION

The cardiovascular system is a complex network comprised of the heart, blood vessels, and lymphatic vessels. These components work together as the driving force behind blood circulation, ensuring the supply of nutrients and oxygen throughout the body while maintaining the water balance [1–3]. Recent evidence indicates that blood and lymphatic vessels are regulated [4–6]. Notably, lymphangiogenesis studies have revealed that lymphatic endothelial cells (LECs) are not solely derived from a subset of blood endothelial cells (BECs) in the cardinal vein (CV) as previously thought [7]. Instead, LECs originate from diverse types of (B)ECs, indicating a more complex origin model [8–13]. Recent studies have highlighted that LECs in the CV are embryologically derived from PAX3(+) cells originating from the lateral plate mesoderm (LPM) [14,15]. Migration of PAX3(+) cells to the CV is regulated by a centrifugal mechanism. However, specific molecular mechanisms underlying this regulation remain unclear. Epigenetic mechanisms are crucial for ensuring proper functioning and maturation of endothelial cells during development and for maintaining the integrity of the cardiovascular system [16,17].

Disruptor of telomeric silencing 1-like (DOT1L) was shown in previous studies performs critical functions during cardiovascular development. DOT1L plays vital roles in cardiac development and function [18–21]. Additionally, DOT1L is particularly important for the development and function of lymphatic vessels (LECs) [18,20,21], and DOT1L was identified as a key protein regulating LECs migration over blood endothelial cell (BECs) migration in a genome-wide gene knockdown study [22]. Our previous study demonstrated that DOT1L plays an important role in the development and function of lymphatics in developing mouse embryos by modulating histone H3K79 methylation and transcription [23]. Furthermore, DOT1L promoted BEC migration, tube formation, and sprout formation *in vitro* and *in vivo*, primarily by regulating VEGFR2 expression [20]. Remarkably, recent research has demonstrated that DOT1L has a different transcriptional regulatory network depending on the type of endothelial cell, whether LEC or BEC [24]. This indicates the complexity of DOT1L function and highlights its significance in cardiovascular development and lymphangiogenesis.

However, whether DOT1L is essential for the function (s) of BECs and/or LECs during adult life remains unclear. In the present study, we generated DOT1L knockout and reporter alleles and analyzed their expression patterns and vascular functions. Our analysis revealed that DOT1L is broadly expressed in various organs of adult mice, and its loss results in brain hemorrhage and increased vascular permeability. Furthermore, DOT1L silencing *in vitro* led to impaired tube formation and migration, likely owing to aberrant transcriptional regulation.

## MATERIALS AND METHODS

### Mouse models and dye injection

All animal studies were reviewed and approved by Institute of Animal Care and Use Committee (IACUC) of Konkuk University (IACUC#KU18027). For conditional or inducible *Dot1l* KO studies, *Dot1l*^2f/2f^; Tie2-cre or *Dot1l*^2f/2f^; ROSA26-creER mice were generated by breeding Tg (Tie2-cre) or ROSA26-creER strains with mice harboring the Dot1l conditional allele (*Dot1l*^2f/2f^) [25]. Tg (Tie2-cre) (stock #004128) and ROSA26-creER (stock #004847) mice were purchased from the Jackson Laboratory. Some *Dot1l*^2f/2f^; Tie2-cre mice were viable beyond postnatal day (PD) 3. Latex dye was injected into the left ventricle of the survived PD 5 mice as previously described [26]. Briefly, the mice were anesthetized, placed ventral side up, and the thoracic cavities were opened. The right atrium was excised. After systemic vessel perfusion with phosphate-buffered saline (PBS), blue latex dye (15 μL/g body weight; VWR International, Radnor, PA, USA) was slowly and gently injected into left ventricle. The gastrointestinal tract was removed, fixed in 4% paraformaldehyde, and subjected to tissue clearing using an organic solvent (benzyl alcohol/benzyl benzoate, 1:1; Sigma-Aldrich, St. Louis, MO, USA).

4-Hydroxytamoxifen (HTM H6278, Sigma) was dissolved in DMSO (2mg/25g) and intraperitoneally injected into 7 to 8 weeks-old *Dot1l*^2f/2f^; ROSA26-creER and control (*Dot1l*^2f/2f^) mice every other day for 2 weeks. One month after the last HTM injection, Evans Blue (EB) dye (1 mL of 3% [in 0.9% saline]/kg of mouse) was injected into the tail vein of *Dot1l*^2f/2f^; ROSA26-creER and control mice. Thirty minutes after EB injection, mice were anesthetized with isoflurane and perfused with 1% paraformaldehyde in 0.05M citrate buffer through the left ventricle. Approximately 4 cm of the small intestine was collected and placed between two pieces of Whatman filter paper for 10 seconds. and weighed. The tissues were then placed in 1 mL of formamide overnight at 56°C to extract EB. The following day, after removing the tissues, the amount of EB extracted was measured using a spectrophotometer (A_620_). Values were expressed as ng EB/mg of tissue.

### Cell culture and DOT1L knockdown

Human umbilical vein endothelial cells (HUVEC; C-12200, PromoCell) were purchased and maintained in Endothelial Cell Basal Medium supplemented with supplements (C-22215, C-39216, and PromoCell). To silence DOT1L in HUVEC, lenti-shDOT1L vectors were constructed, as described in our previous study [27]. Briefly, hDOT1L KD1 sequences (5′-GGCTCTGCGACAAGTACAA-3′ and 5′-TTGTACTTGTCGCAGAGCC-3′) and hDOT1L KD2 sequences (5′-GCCCGCAAGAAGAAGCTAA and 5′-TTAGCTTCTTCTTGCGGGC-3′) were cloned into the BbsI/HindIII sites under the U6 promoter. To generate lentiviral viruses, HEK293T cells were grown in Dulbecco’s modified Eagle’s medium (DMEM) supplemented with 10% fetal bovine serum and 1% penicillin/streptomycin. Once the cells reached ~85% confluency, the lenti-shDOT1L virus vector and packaging vectors (psPAX2 [Addgene # 12260] and pMD2.G [Addgene # 12259] vectors) were transfected into HEK293T cells using the Superfect reagent (Qiagen, Germantown, MD, USA), and the HEK293T cells were maintained in Freestyle 293T media. Supernatants containing viral particles were harvested at 26, 38, and 50h post-transfection and concentrated using an Amicon Ultracell 100K column (Amicon, MilliporeSigma, Burlington, MA, USA). After the production of the lenti-shDOT1L virus, HUVEC were transduced when the cells reached ~50% confluency by using polybrene (10 μg/mL). Transduced HUVECs were then subjected to puromycin (1 μg/mL) selection 48 h after transduction for >7 days.

### Analysis of cell migration and tube formation

For the tube formation assay, the HUVECs were counted and seeded into Matrigel-coated 48-well plates. Approximately 5×10^3^ cells were plated in each well. The plates were imaged 12 h after seeding to assess tube formation. The number of tube branches originating from the center of each well was counted as a measure of angiogenic potential.

For scratch wound closure analysis, approximately 2×10^5^ HUVECs were seeded in a 6-well plate. Once the cells reached approximately 95% confluence, scratches or wounds were created across the cell monolayer using a p200 pipette tip. After 17 h, the wound closure was imaged using a phase-contrast microscope. The distance of wound closure was measured using the ImageJ software, which allowed quantification of the extent of cell migration and wound healing.

### X-gal and hematoxylin and eosin staining

X-gal staining was performed as describe in a previous study [25]. Briefly, internal organs from ~ 6wks-old mice were harvested, fixed for 10 min in PBS containing 1% formaldehyde, 0.2% glutaraldehyde, 2 mM MgCl_2_, 5 mM ethylene glycol tetraacetic acid (EGTA), and 0.02% NP-40, and stained at 37°C overnight with X-gal staining solution (5 mM K4Fe(CN)_6_, 5 mM K3Fe(CN)_6_·3H_2_O, 2 mM MgCl_2_, 0.01% Na-deoxycholate, 0.02% NP-40, 100 mM phosphate buffer (pH 7.3), and 0.75 mg/mL X-gal). The stained organs were fixed with formalin and subjected to tissue clearance in an organic solution (benzyl benzoate:benzyl alcohol [1:1]).

For hematoxylin and eosin (H&E) staining, tissues were dehydrated with differential concentration of ethanol series and xylene, embedded into paraffin, and cut with 6 μm-thickness. Tissue sections were rehydrated with different concentrations of ethanol and H_2_O. They were then stained with eosin for the cytoplasm and hematoxylin for the nuclei, and mounted. Images were captured using an Olympus stereo microscope.

### Reverse transcription quantitative polymerase chain reaction

Total RNAs was extracted using a RNeasy Plus Mini Kit (74104; Qiagen, USA), and cDNA was synthesized using Maxime RT PreMix cDNA synthesis kit (iNtRON; Seongnam, Korea). Quantitative polymerase chain reaction (qPCR) was performed on a StepOnePlus System (Applied Biosystems, Foster City, CA USA) using the Fast SYBR Green Master Mix (4385616; Applied Biosystems, USA). Primer sequences used in the qPCR analysis are presented (Table 1).

### Statistical analysis

All statistical analyses were performed using Prism 8.0.2 (GraphPad Software, San Diego, CA, USA). A t-test was used to determine the statistical significance in tube formation, cell migration, and qPCR assays. Mean values with the standard error of the mean (SEM) are presented as graph errors. Statistical significance was determined at a p-value lower than 0.05 (*), 0.01 (**), 0.001 (***) or NS (not significant).

## RESULTS

### *Dot1l* expression during post-natal life

**Figure 1:**
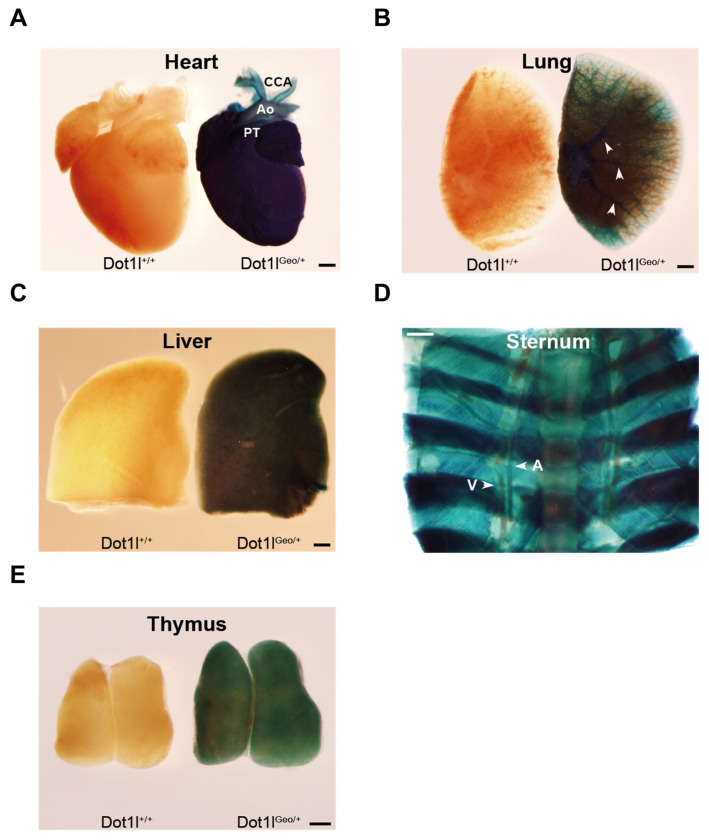
Detection of mouse *Dot1l* expression in postnatal mouse organs using *Dot1l*^Geo/+^ allele. A. X-Gal staining showing expression pattern of *Dot1l* in heart, lungs, liver, sternum and thymus. Arrow head: blood vessels. A, artery. V, vein. CCA, common carotid artery. Ao, aorta. PT, pulmonary trunk. Scale bar = 200 μm.

In this study, we first assessed *Dot1l* expression in adult organs using *Dot1l*^Geo/+^ mice, as previously described in another study [28]. Similar to the embryonic expression pattern, ubiquitous Dot1l expression in adult organs, including the heart, lungs, liver, sternum, and thymus, was detected using X-Gal staining (Figure 1). These results demonstrated that Dot1l is widely expressed in various organs during adulthood, mirroring its embryonic expression pattern.

### Analysis of postnatal vessel phenotypes in Dot1l knockout mice

**Figure 2:**
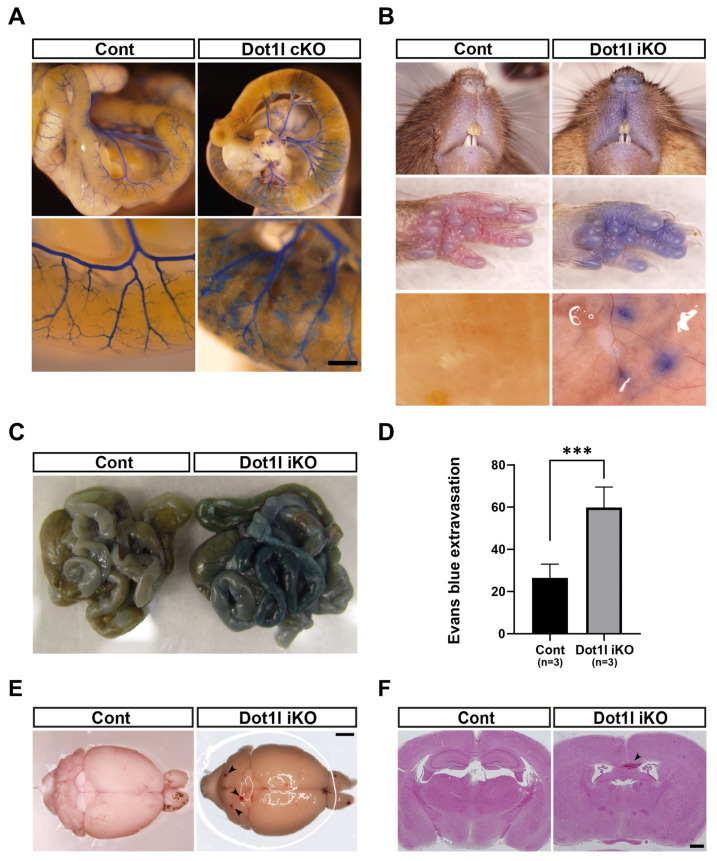
Phenotypic analysis of *Dot1l* knockout (KO) strains. (A) Visualization of latex dye-injected *Dot1l* cKO intestine. Note that a diffused vessel structure was detected in the *Dot1l* cKO intestine. (B) Representative images of Evans blue dye leakages in face, dorsal skin and intestine. (C) Representative images of Evans blue dye-injected intestines. (D) Quantification of leaked Evans blue dye extracted from intestine. Graphs represent the mean and standard error mean. ***, p<0.001. (E) Representative photographs of hemorrhage-like spots in the inducible *Dot1l* KO brain. Arrow head: hemorrhagic spot(s). (F) Hematoxylin and eosin (H&E) staining in the inducible *Dot1l* KO brain. Arrow head: hemorrhage. Scale bar = 200 μm.

In our previous study [23], we demonstrated that most conditional Dot1l knockout (cKO) mice using the Tg (Tie2-cre) strain exhibited late gestational embryonic lethality due to lymphatic anomalies such as blood-mixing, lymphatic hypoplasia, and chylous ascites. However, few cKO mice are viable after birth and display lymphatic phenotypes. Viable cKO mice were used to assay vessel phenotypes after birth. On approximately PD5, these mice were injected with latex dye into the left ventricle, which revealed a focal diffuse and nodular pattern of dye distribution around the hemorrhage-like spots of the intestinal tubes. To further examine DOT1L function during adulthood, we employed inducible knockout (iKO) mice using the ROSA26-creER strain. As depicted in (Figure 2E, F) hemorrhagic spots were found in iKO brains and increased vascular permeability was observed in these mice.

### Dot1l depletion impairs endothelial cells migration, tube formation, and expression of vascular genes

**Figure 3:**
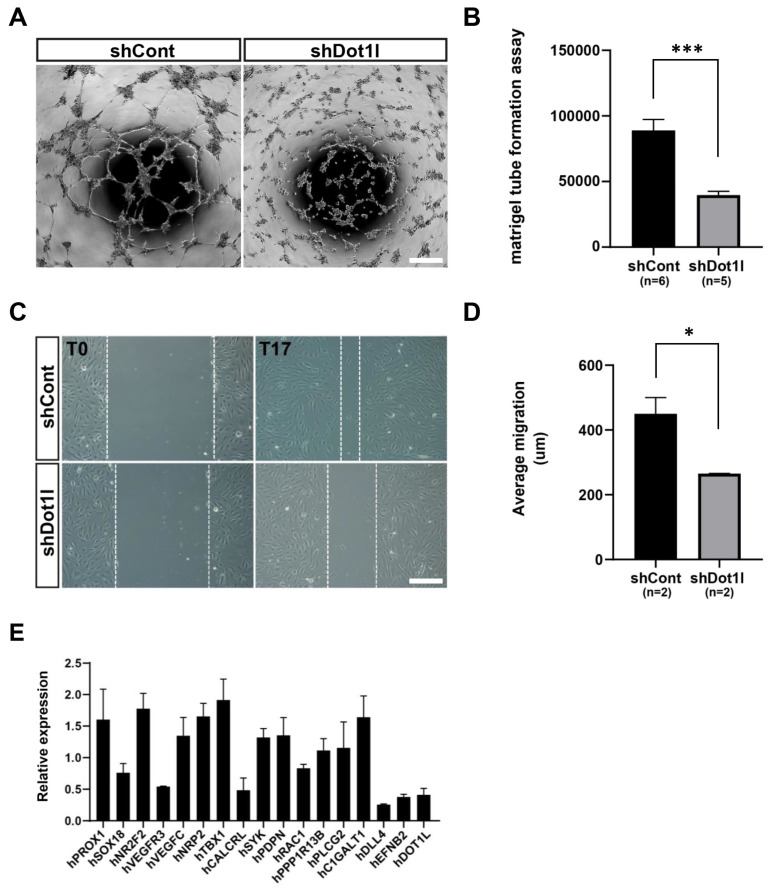
Analysis of cellular effects of DOT1L silencing in HUVECs. (A) Assay of in vitro tube formation in DOT1L-knock down HUVECs, and quantification (B). (C) Assay of cell migration in the DOT1L-knock down HUVECs, and quantification (D). Graphs in (B) and (D) represent the mean and standard error mean. * p<0.05. *** p<0.001. (E) qPCR analysis of vascular gene expression in the DOT1L-knock down HUVECs. Scale bar = 200 μm. HUVECs, human umbilical vein endothelial cells; RT-qPCR, reverse transcription quantitative polymerase chain reaction.

To investigate Dot1l function in the regulation of vessel formation, *in vitro* tube formation and cell migration assays were performed. The results shown in (Figure 3A, C) indicate a significant reduction in the capacity for both tube formation and cell migration in DOT1L-depleted HUVECs. Next, we examined the gene expression patterns in DOT1L-depleted HUVECs. As shown in (Figure 3E), a subset of the genes examined, including *PROX1*, *SOX18*, *NR2F2*, *VEGFR3*, *VEGFC*, *NRP2*, *TBX1*, *CALCRL*, *SYK*, *PDPN*, *RAC1*, *PPP1R13B*, *PLCG2*, *C1GALT1*, *DLL4*, *EFNB2* and *DOT1L* were repressed in KD HUVECs.

## DISCUSSION

During embryonic development, precise control of gene expression is essential for the formation of various tissues and organs [29,30]. Various signaling pathways and transcription factors regulate the expression of specific genes at different stages of development [31,32]. Epigenetic modifications, such as histone methylation, play a key role in this process by regulating chromatin structure and accessibility, thereby influencing gene expression during cardiovascular development and function [16,33,34]. The present study examined DOT1L function in adult mice and found that DOT1L loss induced cerebrovascular hemorrhage and increased vascular permeability. In addition, *in vitro* experiments showed that deletion of DOT1L impaired tube formation and migration in HUVECs. Furthermore, they found that this was due to the transcriptional regulation by DOT1L. As expected, *Dot1l* was widely expressed in the cells of various organs, and this expression pattern was likely associated with essential DOT1L functions.

Previous studies have shown that DOT1L is essential for cardiac development and function [19,35,36]. DOT1L regulates the expression of crucial genes that control cardiomyocyte proliferation, differentiation, and cardiac morphogenesis. Studies in mice have shown that DOT1L is necessary for proper heart formation, and its deficiency leads to severe cardiac defects, including thinning of the ventricular walls, improper chamber formation, and abnormal cardiac function [37]. DOT1L loss in cardiomyocytes leads to dilated cardiomyopathy due to the aberrant expression of genes essential for heart function [19]. In addition, Cattaneo et al [36] showed that DOT1L specifically regulates gene sets critical for left ventricle formation and postnatal cell cycle withdrawal in cardiomyocytes via H3K79 methylation.

Our analysis revealed that DOT1L loss in adults leads to increased vascular permeability and eventual cerebrovascular bleeding. Proper regulation of vascular permeability is crucial for tissue homeostasis and immune responses, and dysregulation can lead to pathological conditions such as edema and inflammation [38–40]. Similarly, histone methylation plays a critical role in regulating vascular integrity, which is essential for maintaining the barrier function of blood vessels and preventing vascular leakage and inflammation. Epigenetic modification of histones, particularly methylation, can affect the expression of genes involved in endothelial cell adhesion, junctional complexes, and cytoskeletal organization, thereby influencing the overall vascular integrity [41–43]. For example, histone methyltransferase Suv39h1 repress eNOS transcription via binding to its proximal promoter, in which reduced H3K9me3 level induces IFN-γ-induced eNOS repression [44]. In general, eNOS is critical for the regulation of endothelial function and vascular tone [45,46]. Jmjd3, a histone H3K27me3 demethylase, expression is upregulated upon oxygen-glucose deprivation/reperfusion injury in endothelial cells, in which the elevation of Jmjd3 leads to increase its interactions with Nf-κb (p65/p50) and CCAAT-enhancer-binding protein β in interleukin 6 (Il-6) gene promoter, and then decrease H3K27me3 levels to promote Il-6 expression [47]. In addition, Jmjd3 expression is enhanced in endothelial cells after lipopolysaccharide treatment to activate target gene expression via demethylation of H3K27me3 [48]. Overexpression of vascular endothelial cadherins increases claudin-5 expression by preventing the binding of PRC2 to the *CLDN5* gene [49]. Recently, it was shown that common and/or diverse gene signatures and epigenetic landscapes exist across organotypic BECs in mice [34].

Consistent with our analysis, silencing DOT1L in HUVECs leads to cell death and reduced capillary sprout formation, which are associated with altered H3K79 methylation and transcription factor ETS-1 binding, resulting in the regulation of VEGFR2 expression [20]. Our previous study revealed that DOT1L regulates distinct sets of transcripts in different types of endothelial cells, likely through H3K79 methylation [24]. One of these gene sets included cell-cell adhesion molecules (CLDN1, CLDN11, and PCDHs), which were found to be differentially expressed by DOT1L depletion or overexpression. Consequently, it is plausible that the cerebrovascular hemorrhage observed in the induced DOT1L KO mice was due to the repressed expression of these cell-cell adhesion molecules.

Our study, along with others, showed that DOT1L plays a crucial role in maintaining vessel integrity in adults through transcriptional regulation. Cerebrovascular hemorrhage observed in DOT1L KO mouse could potentially open new avenues for utilizing DOT1L in the treatment of cerebral hemorrhage. However, further research is required to explore this issue in more detail and gain a deeper understanding of the therapeutic potential of DOT1L in the treatment of cerebral hemorrhage.

## Figures and Tables

**Table 1:** Primer and knockdown (KD) sequences used in this study

Genes	Primer sequence (5′–3′)	Accession No.	Size (bp)
*hPROX1*	F TGAATCCCCAAGGTTCTGAG	NM_002763	108
	R CATACGAGTTCGCCCTCTTC		
*hSOX18*	F AGCGTGGAAGGAGCTGAAC	NM_018419	105
	R GGCCGGTACTTGTAGTTGG		
*hNR2F2*	F CGGATCTTCCAAGAGCAAGT	NM_021005	100
	R AGGCATCTGAGGTGAACAGG		
*hVEGFR3*	F GTACATGCCAACGACACAGG	NM_002020	97
	R TCACGAACACGTAGGAGCTG		
*hVEGFC*	F GTCGCGACAAACACCTTCTT	NM_005429	105
	R GTAGCTCGTGCTGGTGTTCA		
*hNRP2*	F GAAGAGGAGGCCACAGAGTG	NM_201266	99
	R CTCGAGGAAATCGAAGTTGC		
*hTBX1*	F GTTTCCCACCTTCCAAGTGA	NM_080647	99
	R TACCGGTAGCGCTTATCGTC		
*hCALCRL*	F GCAGCTCTGCCCTGATTACT	NM_005795	103
	R TCTGTTGCTTGCTGGATGTC		
*hSYK*	F AAGCAAATGTCATGCAGCAG	NM_003177	103
	R CCAAGTTCTGCCATCTCCAT		
*hPDPN*	F ATTTTCCCCCAGCTCAGAAT	NM_006474	101
	R CTTCCCAAAACGAAGAGCAG		
*hRAC1*	F AACCAATGCATTTCCTGGAG	NM_006908	99
	R TCCCATAAGCCCAGATTCAC		
*hPPP1R13B*	F CGTTTTACCTTCGGGTTCAA	NM_015316	96
	R CTTTCTGAAGGTGGCTGAGG		
*hPLCG2*	F TGACAAGATCGAGGGCTTCT	NM_002661	102
	R TCTTTCTGGCGAACTGCTTT		
*hC1GALT1*	F ATCCCTTTGTGCCAGAACAC	NM_020156	103
	R CAGCAACCAGGACCCTCTAC		
*hDLL4*	F TGCAGGAGTTCATCAACGAG	NM_019074	96
	R GGAAGTGCTTAAGGCAGACG		
*hEFNB2*	F TTCCGAAGTGGCCTTATTTG	NM_004093	95
	R TCCGGTACTTCAGCAAGAGG		
*hDOT1L*	F CACATTGGAGAGAGGCGATT	NM_032482	104
	R GATCCACCTCAGGACCAAAG		

*PROX1*, prospero homeobox 1; *SOX18*, SRY-box transcription factor 18; *NR2F*, nuclear receptor subfamily 2 group F member 2; *VEGFR3*, vascular endothelial growth factor receptor 3; *VEGFC*, vascular endothelial growth factor C; *NRP2*, neuropilin 2; *TBX1*, T-box transcription factor 1; *CALCRL*, calcitonin receptor like receptor; *SYK*, spleen associated tyrosine kinase; *PDPN*, podoplanin; *RAC1*, Rac family small GTPase 1; *PPP1R13B*, protein phosphatase 1 regulatory subunit 13B; *PLCG2*, phospholipase C gamma 2; *C1GALT1*, core 1 synthase, glycoprotein-N-acetylgalactosamine 3-beta-galactosyltransferase 1; *DLL4*, delta like canonical Notch ligand 4; *EFNB2*, ephrin B2; *DOT1L*, DOT1-like histone lysine methyltransferase.
